# Genetic diversity of a *Silybum marianum* (L.) Gaertn. germplasm collection revealed by DNA Diversity Array Technology (DArTseq)

**DOI:** 10.1371/journal.pone.0308368

**Published:** 2024-08-07

**Authors:** Damiano Puglisi, Marianna Pasquariello, Tommaso Martinelli, Roberta Paris, Pasquale De Vita, Nicola Pecchioni, Salvatore Esposito, Laura Bassolino

**Affiliations:** 1 Council for Agricultural Research and Economics, Research Centre for Cereal and Industrial Crops (CREA-CI), Foggia, Italy; 2 NBFC, National Biodiversity Future Center, Piazza Marina, Palermo, Italy; 3 Council for Agricultural Research and Economics, Research Centre for Cereal and Industrial Crops (CREA-CI), Bologna, Italy; 4 Council for Agricultural Research and Economics, Research Centre for Plant Protection and Certification (CREA-DC), Firenze, Italy; KGUT: Graduate University of Advanced Technology, ISLAMIC REPUBLIC OF IRAN

## Abstract

*Silybum marianum* (L.) Gaertn. is a multipurpose crop native to the Mediterranean and middle east regions and mainly known for the hepatoprotective properties of fruit-derived silymarin. Despite growing interest in milk thistle as a versatile crop with medicinal value, its potential in agroindustry is hindered by incomplete domestication and limited genomic knowledge, impeding the development of competitive breeding programs. The present study aimed to evaluate genetic diversity in a panel of *S*. *marianum* accessions (n = 31), previously characterized for morphological and phytochemical traits, using 5,178 polymorphic DArTseq SNP markers. The genetic structure investigated using both parametric and non-parametric approaches (e.g. PCA, AWclust, Admixture), revealed three distinctive groups reflecting geographical origins. Indeed, Pop1 grouped accessions from Central Europe and UK, Pop3 consisted mainly of accessions of Italian origin, and Pop2 included accessions from different geographical areas. Interestingly, Italian genotypes showed a divergent phenotypic distribution, particularly in fruit oleic and linoleic acid content, compared to the other two groups. Genetic differentiation among the three groups, investigated by computing pairwise fixation index (F_ST_), confirmed a greater differentiation of Pop3 compared to other subpopulations, also based on other diversity indices (e.g. private alleles, heterozygosity). Finally, 22 markers were declared as putatively under natural selection, of which seven significantly affected some important phenotypic traits such as oleic, arachidonic, behenic and linoleic acid content. These findings suggest that these markers, and overall, the seven SNP markers identified within Pop3, could be exploited in specific breeding programs, potentially aimed at diversifying the use of milk thistle. Indeed, incorporating genetic material from Pop3 haplotypes carrying the selected loci into milk thistle breeding populations might be the basis for developing milk thistle lines with higher levels of oleic, arachidonic, and behenic acids, and lower levels of linoleic acid, paving new avenues for enhancing the nutritional and agronomic characteristics of milk thistle.

## Introduction

Milk thistle (*Silybum marianum* (L.) Gaertn.) is an annual or biannual species belonging to the Asteraceae family, native to southern Europe, Asia Minor, and Northern Africa. The species is diploid (2n = 34) [1, 2], with a genome of ~ 694.4 Mb [3], mainly autogamous with an average outcrossing rate of 2–4% under field conditions [4].

The genus *Silybum* was described to group only two species: *S*. *marianum* and *S*. *eburneum* [1] and it was argued that probably the two forms are only variants of the same species [4], although this classification is still under debate [5, 6]. Milk thistle has been utilized for more than 2000 years and mainly cultivated in Asia and Eastern Europe as a medicinal plant due to the phytochemical properties of its prominent compound namely silymarin, a complex of bioactive flavonolignans accumulated in the seed integument from 1.5% up to 4.3% of the total fruit weight [7, 8]. Pharmacologically relevant actions of silymarin include hepatoprotective properties and antioxidant, anti-inflammatory, antifibrotic, hypolipidemic, neurotrophic, and neuroprotective effects [9]. Besides silymarin, fruits are also rich in oil and protein, showing that milk thistle can also have different possible agrifood and industrial applications [10]. From an agronomic perspective, milk thistle is characterized by significant fruit and plant biomass yield and its potential use for fodder, bioenergy production and phytoremediation as well as for feed and cosmetics is relatively unexplored [11–13].

Despite the increasing interest in *S*. *marianum* as a multipurpose crop and its recognised importance as a medicinal species, its exploitation in agroindustry systems is mainly limited by the fact that the species is not completely domesticated [6, 14] and the genomic knowledge is still very poor to start a breeding program. In addition, a comprehensive understanding of the genetic variability and relationships between accessions in the available germplasm collections represents a key step in biodiversity conservation, monitoring, and exploitation [15] and thus, a crucial step toward an efficient breeding program design. Many molecular marker technologies have been developed and applied to study genetic diversity in germplasm collections and breeding programs [16], including RFLPs [17], RAPDs [18], ISSRs [19], SSRs [20] and AFLPs [21]. There have been some recent attempts to investigate genetic diversity in different *S*. *marianum* collections using the SCoT (Start Codon-Targeted) [22], AFLP (Amplified Fragment Length Polymorphism) [23], ISSR (Inter Simple Sequence Repeats) [24], and co-dominant insertion/deletion (InDel) [25] markers. However, their major limitations are poor genome coverage, low discrimination ability, poor reproducibility, and technical and time requirements, together with high cost per unit, making them unsuitable for high-throughput genotyping. Moreover, all these studies have mainly investigated the genetic variation of a few accessions, coming from some well-defined geographical areas such as Iran [22–24] or Korea [25].

DArT (Diversity Array Technology Pty Ltd) markers are a prime alternative as they combine high-throughput DNA array technology, restriction site polymorphism analysis, genome complexity reduction, and PCR amplification leading to the production of thousands of polymorphic loci in a single assay [26] thus providing a cost-effective and efficient means for plant genotyping [27–33], even in species where genome sequence information are not available [34, 35]. DArT markers have been applied successfully in genomic studies in many species including those with large and complex genomes such as barley [36], sugarcane [37], wheat [38], rye [39], oat [40], and strawberry [33]. However, while the initial DArT implementation on the microarray platform involved fluorescent labeling of representations and hybridization to dedicated DArT arrays, currently the DArTseq method deploys efficient genotyping-by-sequencing platforms which allows genome-wide marker discovery through restriction enzyme-mediated genome complexity reduction and sequencing of the restriction fragments [34].

In this study, a DArTseq approach was applied to assess the genetic diversity of 31 *S*. *marianum* accessions from Southern Europe (e.g., Italy, Spain), Central Europe and UK (e.g., Austria, Germany), and other countries worldwide (e.g., Canada, North Korea) (Table 1), providing valuable insights into milk thistle diversity collected across different continents and climates. The collection was previously characterized for phenotypic traits including fruit morphology and chemical traits such as flavonolignans and fatty acids content [5, 41]. The comparison between the genotypic and phenotypic data conducted in the present study aimed firstly to better understand the origin of the accessions preserved in the germplasm bank and their botanical classification, but also to identify interesting genetic material to be used in milk thistle breeding programs.

**Table 1:** List of *S*. *marianum* accessions used in the present study. Accession number, DArT sample code, Accession origin: ISO code of the country where the accession was originally collected; Species; Accession description; Donor code: FAO code of donor institutions; and donor accession number were shown.

Accession number	DArT sample code	Accession origin	Species	Accession description	Donor code	Donor accession number
**G1**	a1, a1bis, a2, a3	-	*S*. *marianum*	-	DEU146^a^	SIL1
**G2**	a4, a5, a6	-	*S*. *marianum*	-	DEU146^a^	SIL2
**G3**	a7, a8, a9	North Korea	*S*. *marianum*	-	DEU146^a^	SIL4
**G4**	a10, a11, a12	-	*S*. *marianum*	Wild	DEU146^a^	SIL8
**G5**	a13, a14, a15	-	*S*. *marianum*	Wild	DEU146^a^	SIL9
**G6**	a16, a17, a18	-	*S*. *marianum*	Wild	DEU146^a^	SIL10
**G7**	a19, a20, a21	Austria	*S*. *marianum*	Traditional cv./landrace	AUT001^b^	BVAL901047
**G8**	a22, a23, a24	Romania	*S*. *marianum*	De Prahova	AUT001^b^	BVAL901578
**G9**	a25, a26, a27	Hungary	*S*. *marianum*	“Fehér”	HUN003^c^	RCAT040358
**G10**	a28, a29, a30	Germany	*S*. *marianum*	-	HUN003^c^	RCAT074067
**G11**	a31, a32, a33	United Kingdom	*S*. *marianum*	-	HUN003^c^	RCAT071128
**G12**	a34, a35, a36	Spain	*S*. *marianum*	-	HUN003^c^	RCAT074546
**G13**	a37, a38, a39	Czech Republic	*S*. *marianum*	-	HUN003^c^	RCAT071195
**G14**	a40, a41, a42	Germany	*S*. *marianum*	-	HUN003^c^	RCAT077005
**G15**	a43, a44, a45	Germany	*S*. *marianum*	-	HUN003^c^	RCAT040360
**G16**	a46, a47, a48	Poland	*S*. *marianum*	-	HUN003^c^	RCAT040357
**G17**	a49, a50, a51	Canada	*S*. *marianum*	-	HUN003^c^	RCAT069989
**G18**	a52, a53, a54	Belgium	*S*. *marianum*	-	HUN003^c^	RCAT074006
**G19**	a55, a56, a57	Czech Republic	*S*. *marianum*	-	HUN003^c^	RCAT057474
**G20**	a58, a59, a60	Germany	*S*. *marianum*	-	HUN003^c^	RCAT057475
**G21**	a61, a62, a63	Poland	*S*. *marianum*	-	HUN003^c^	RCAT040356
**G22**	a64, a66	Italy	*S*. *marianum*	Wild	-	-
**G23**	a67, a68, a69	Italy	*S*. *marianum*	Wild	-	-
**G24**	a70, a71, a72	Italy	*S*. *marianum*	Wild	Siena botanical garden	648
**G25**	a73, a74, a75	Italy	*S*. *marianum*	Wild	Naples botanical garden	114
**G26**	a76, a77, a78	Italy	*S*. *marianum*	Wild	-	-
**G31**	a79, a80, a81	Italy	*S*. *marianum*	Wild	-	-
**G33**	a82, a83, a84	Hungary	*S*. *marianum*	"Minardi"	-	-
**G34**	a85, a86, a87	Italy	*S*. *marianum*	Wild	-	-
**G35**	a88, a89, a90	Italy	*S*. *marianum*	Wild	-	-
**SIL3**	a91, a92, a93, a93bis	Germany	*S*. *eburneum*	Wild	DEU146^a^	SIL3

^a^IPK, Gatersleben, Germany

^b^AGES, Linz, Austria

^c^Nébih, Tápiószele, Hungary

## Materials and methods

### Plant material

**Fig 1:**
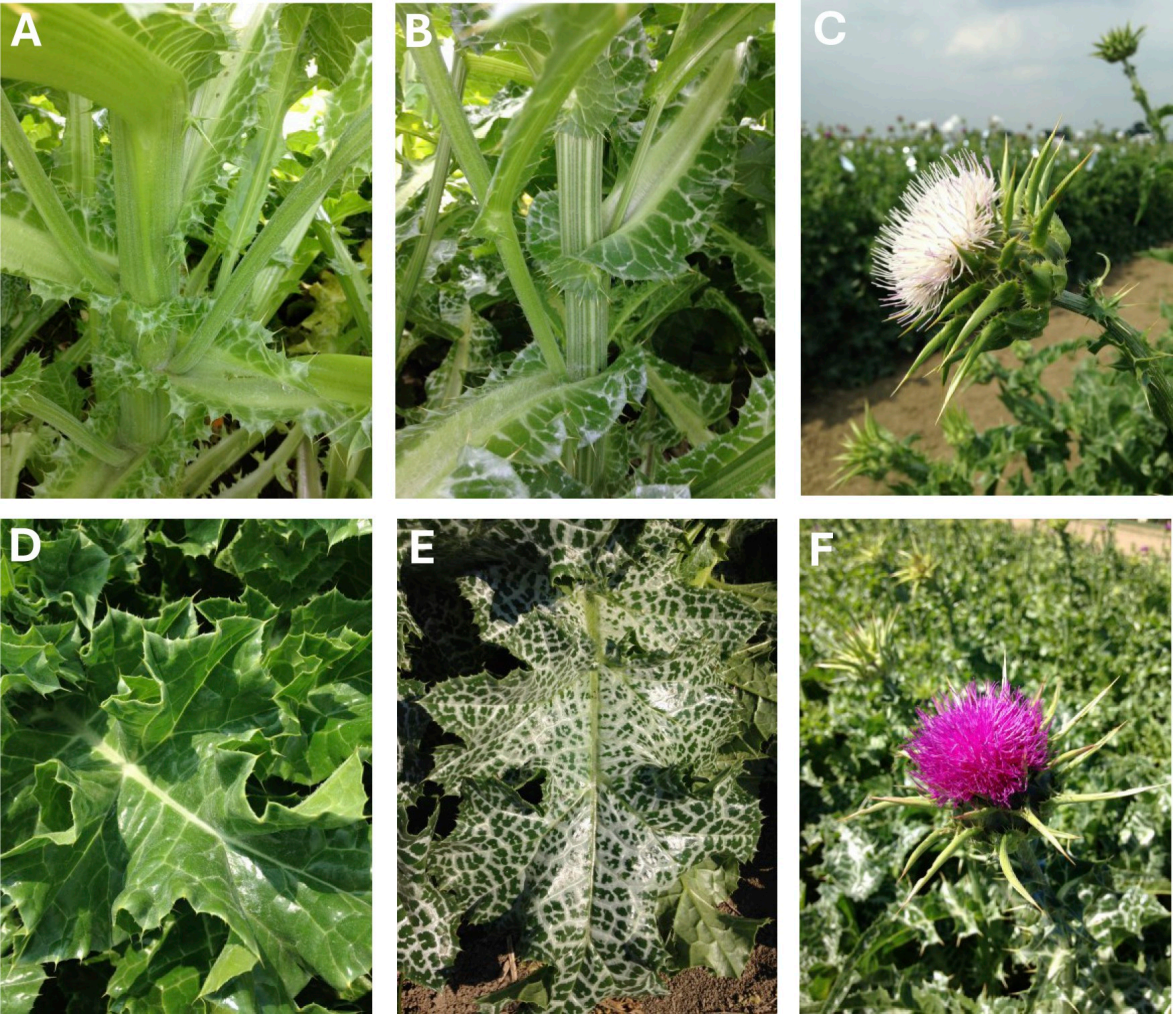
Leaf, stem and flower biodiversity of the *ex-situ Silybum marianum* used in this study. **A)** G20 stem. **B)** G8 striated stem. C) G9 white inflorescence D). G5 non variegated leaf, E) G20 purple inflorescence.

The collection used in the present work comprises 31 accessions of *S*. *marianum* (Table 1 and Fig 1) including accessions of different origins and/or coming from various international germplasm banks. This collection, selected based on its geographical distribution to ensure broad genetic diversity, is stored as seed at CREA-CI (Bologna, Italy) under controlled environmental conditions (-20°C) and moisture content between 3% and 7%. Twenty-six out of 31 accessions (G1-G26) were previously characterized at morphological and phytochemical levels by Martinelli et al [5]. Ten (10) accessions were collected in Southern Europe (Italy: G22, G23, G24, G25, G26, G31, G34 and G35; Spain: G12 and Romania: G8), 14 accessions are from Central Europe and UK (Austria: G7; Belgium: G18; Czech Republic: G13 and G19; Germany: G10, G14, G15, G20 and SIL3, United Kingdom: G11; Hungary: G9 and G33; Poland: G16 and G21), two accessions are from other countries (North Korea: G3 and Canada: G17) and five have unknown origins (Table 1).

The collection also includes an *S*. *eburneum* accession (SIL3) coming from the Leibniz Institute of Plant Genetics and Crop Plant Research (IPK, Gatersleben, Germany; FAO code DEU146) (Table 1).

### DNA extraction and DArT sequencing

Given that, a large part of the collection included accessions collected from the wild and their genetic diversity is still unknown, DNA extraction and DArT analysis were performed on three seedlings for each of 31 accessions, except SIL3 and G1, for which 4 plants were collected, and G22 with two plants, for a total of 94 samples. DNA extraction was performed as described by Martinelli et al. [14]. Each seedling is identified by a two-digit alphanumerical code; correspondence between seedling and accession is listed in Table 1. DNA samples were then processed at Diversity Array Technology (DArT) Pty, Ltd., Bruce, Australia (http://www.diversityarrays.com/). A *PstI-MseI* genome complexity reduction method was used, and a series of digestion-ligation reactions were performed using the protocol described by [26] with some modifications. Both *PstI*- and *MseI*- adaptors were designed to include an Illumina flowcell attachment sequence and only *PstI-MseI* fragments were then amplified on a 30 cycles PCR reaction. Equimolar amounts of amplified products of each sample were then transferred on a 96-well plate, applied to a c-Bot system (Illumina) for a bridge PCR amplification and finally sequenced on an Illumina Hiseq2500 (Illumina Inc., USA) for 77 cycles.

Roughly 5,200 DArTseq markers scoring was achieved using the DArTsoft14 software plugin in the KDCompute application (http://www.kddart.org/kdcompute.html). Two types of DArTseq markers, SilicoDArT markers and SNP markers were both scored by the provider as binary for the presence/absence (1 and 0, respectively) of the restriction fragment with the marker sequence in the genomic representation of the sample. Raw data are available in FIGSHARE database with the following doi: 10.6084/m9.figshare.25551132.

SilicoDArT markers were aligned to the *S*. *marianum* reference genome [3], to identify chromosome positions by BLAST tool and retrieving only hits with identity and alignment length > 95%. SNPs with unknown positions were filtered out.

### Genetic structure, diversity, and identification of outliers SNPs

The genetic structure of the *Silybum* core collection was investigated by various methods for comparison. To have a first description of the data, a Principal Component Analysis (PCA) was performed using GAPIT3 [42] package in R, after filtering away non-polymorphic SNPs. PCA plots were created in R using the ggplot2 package [43]. Then, population structure was inferred using the non-parametric method available in the AWclust software [44]. To cluster individuals in the ASD (Allele Sharing Distance) matrix, AWclust applies Ward’s minimum-variance cluster analysis (R square = D2), where it calculates the genetic distance between every pair of individuals. With the Gap statistics frame, AWclust also estimates the optimal number of groups (K) based on the sample genetic relatedness [45]. Finally, Admixture version 1.23 [46] was used to define the population structure using the following parameters: 10-fold Cross-Validation (CV) for subpopulations (K) ranging from K = 1 to 16 and 1,000 bootstrap replicates. CV scores were used to determine the best K value. Each genotype was assigned to a specific group when the membership coefficient (qi) was higher than 0.60, whereas individuals with qi lower than 0.5 at each K were considered as admixed. Pairwise genetic distance between subpopulations was estimated using Weir and Cockerham’s average F_ST_ using Plink [47]. Nei’s gene diversity (H), Shannon Index (I), and the percentage of private alleles were estimated using Genalex v.6.5 [48].

Signatures of selection were identified using Bayescan 1.2 [49] with 20 pilot runs, 10,000 iterations, a prior odds value of 10, a thinning interval of 10, and a false discovery rate (FDR q-value) < 0.05. Candidate genes overlapping with outliers DArTs were retrieved using bedtools intersect [50] and the *S*. *marianum* reference genome [3]. The gene function was inferred through the Blast analysis (https://blast.ncbi.nlm.nih.gov/Blast.cgi) using the CDS sequences of the *C*. *cardunculus* genome (GCF_001531365.2) as queries.

### Phenotypic differentiation based on population structure and divergent DArT

Phenotypic data previously reported by Martinelli et al. [5, 41] for the same collection investigated here were downloaded and grouped based on the genetic structure identified in this study (S1 Table). For each group, means and variance distributions for each phenotypic trait were calculated and significant differences were assessed using a pairwise T-test implemented in the R environment [51]. Similarly, the allelic effect of DArTs under natural selection identified by Bayescan 1.2 [49] was investigated. In particular, we divided the collection into two groups according to the genotypic profile at each marker to test whether the mean of phenotypic traits was significantly different (T-test; p-value ≤ 0.01).

## Results

### Germplasm collection and genetic characterization

**Fig 2:**
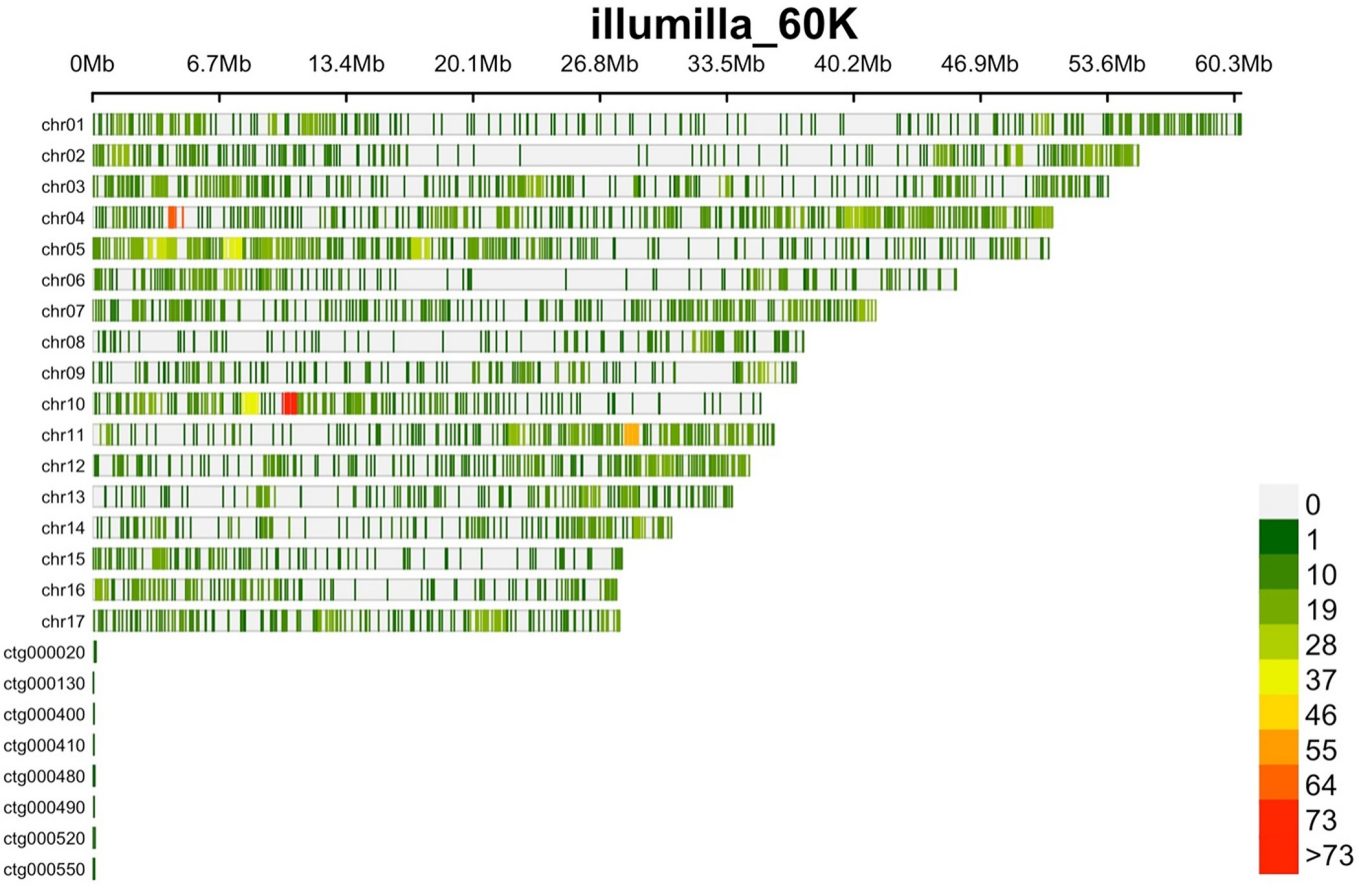
SNP density plot showing the number of variants within 1 Mb window size along the *S*. *marianum* genome. The horizontal axis shows the chromosome length (Mb); the different colour depicts SNP density.

Out of roughly 5,200 DArTseq received from Dart Pty Ltd, 3,629 were mapped in unique regions of the 17 *S*. *marianum* chromosomes [3] (Fig 1), whereas 386 were defined as multi-mapped, for a total of 5,178 polymorphisms. DArTs were distributed across all chromosomes, ranging from 148 on chromosome 15 to 567 on chromosome 4, with an average of 300 DArTs sequences/chromosome (Fig 2). Seventeen additional probes were instead located on eight contigs, with ctg000020 and ctg000550 harboring the highest number (four DArT) and ctg000400, ctg000410 and ctg000490 the lowest (S2 Table).

### Population structure analysis and genetic diversity

**Fig 3:**
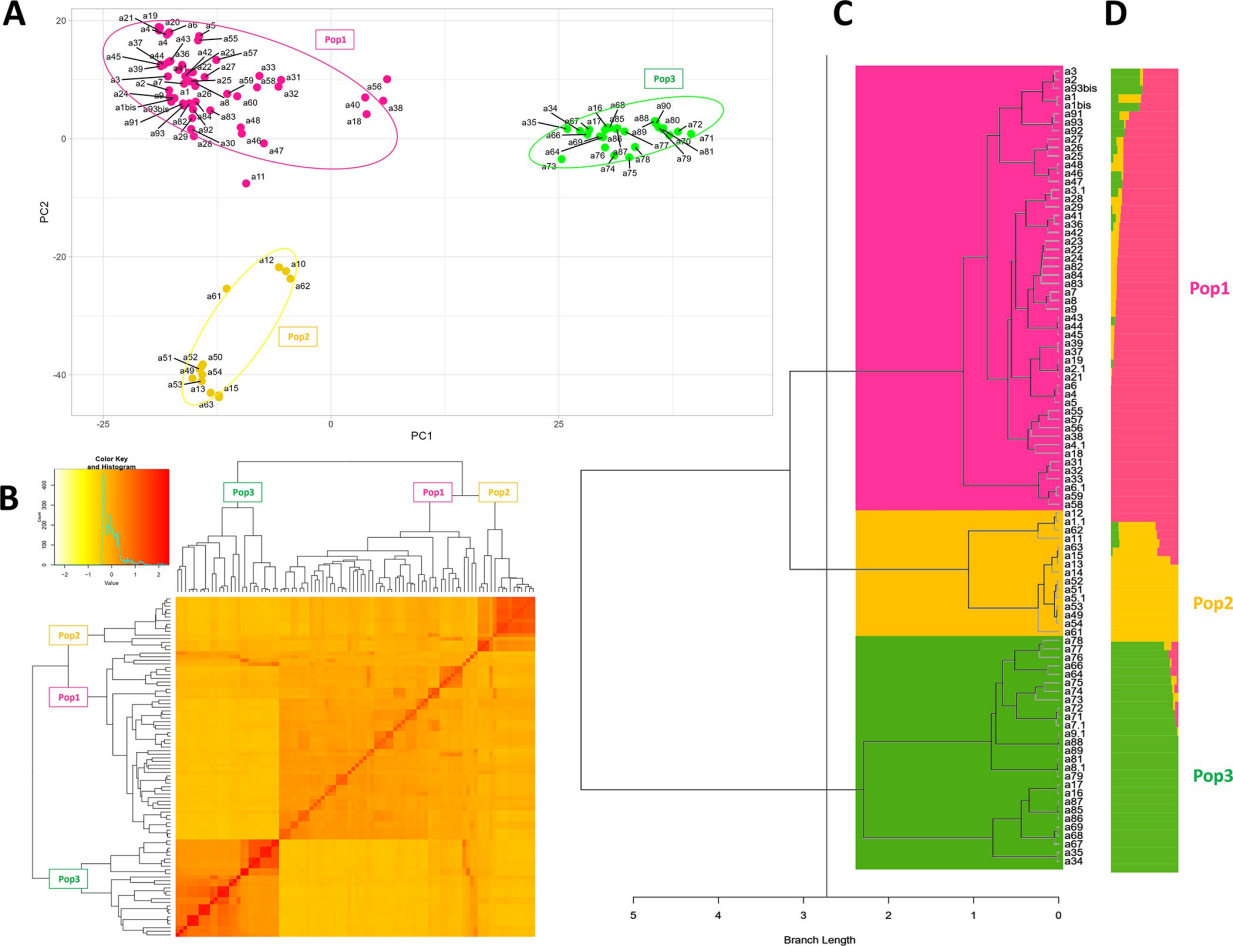
Population structure of *Silybum* accessions using DArTseq technology. **A)** Principal Component Analysis (PCA) using high-quality SNP markers. Samples are colored based on their grouping. **B)** Heat map of kinship matrix created using GAPIT3 [42]. The color histogram indicates the distribution of coefficients of co-ancestry, with the stronger red color showing individuals more related to each other. **C)** Dendrogram obtained through nonparametric hierarchical clustering**. D)** bar-plot describing the population Admixture by the Bayesian approach. Each individual is represented by a thin horizontal line, which is partitioned into K-colored segments whose length is proportional to the estimated membership coefficient (q). The population was divided into three (K = 3) groups according to the most informative K value. The colors indicate the accession membership to the groups identified with the Bayesian analysis.

The genetic structure of *S*. *marianum ex-situ* collection was investigated using both parametric and non-parametric approaches, considering 94 samples of the 31 accessions deposited in the GenBank. Principal Component Analysis (PCA) was used to visualize the genetic variability of the entire dataset (Fig 3A). The first two principal components (PCs) explained 21% of the observed genetic variation and divided the individuals into three different groups (Fig 3A). The first and most abundant group (Pop1) consisted of 53 samples: 12 accessions from Germany (G10, G14, G15 and G20), 6 from Czech Republic (G13 and G19), 18 from Hungary (G9), Poland (G16), Ukraine (G3), United Kingdom (G11), Austria (G7) and Romania (G8); one from Spain (G12-a36); 12 accessions with unknown origin, 10 of witch named G1, G2 and G33 and two named G4 (a11) and G6 (a18); and the only four *S*. *eburneum* accessions (SIL3) of the germplasm collection analyzed in this study. The second group (Pop2) included a total of 14 samples: 9 accessions from Canada (G17), Belgium (G18) and Poland (G21) plus five with unknown origins (G4 and G5), whereas the last group (Pop3) contained 27 samples (24 from Italy: G22, G23, G24, G25, G26, G31, G34 and G35; and two from Spain: G12), plus 1 accession with unknown origin (G6). Worth noting that in some instances (G4, G6, G12, G13, G14, and G19), samples derived from different plants belonging to the same accession clustered in different groups. For example, two G12 plants (a31 and a32) clustered in Pop3, whereas another (a33) was in Pop1. Similarly, two samples from G4 were in Pop2 (a10 and a12), whereas another (a11) was in Pop1.

Similarly, kinship analysis revealed three clusters, perfectly consistent with PCA populations (Fig 3B). AWclust [44] (Fig 3C) and Admixture [46] (Fig 3D) analyses also supported the population structure as described by the PCA plot and kinship, confirming that the germplasm collection in this study could be divided into three clusters (K  =  3), probably reflecting their geographical origin. Indeed, Pop1 contained accessions from Central Europe and UK, Pop3 was mainly constituted by Southern Europe-derived accessions, mainly from Italy and Pop2 included accessions from different regions, such as Canada, Poland, and Belgium.

Genetic differentiation among the three identified groups (Pop1, Pop2, and Pop3) was investigated by computing pairwise fixation index (F_ST_) values. Our findings showed that the genetic differentiation was low between Pop1 and Pop2 (F_ST_ = 0.36) and Pop1 and Pop3 (F_ST_ = 0.37), and higher between Pop2 and Pop3 (F_ST_ = 0.45) (Fig 3), while Nei’s gene diversity (H) and Shannon Index (I) were 0.17 and 0.27, respectively (S3 Table). A higher percentage of private alleles and expected heterozygosity was also detected in Pop3 (0.16% of private alleles and 0.25 of expected heterozygosity) compared with other subpopulations. Specifically, Pop3 exhibited 0.07% and 0.01% of private alleles and an expected heterozygosity of 0.17 and 0.09 greater than Pop1 and Pop2, respectively (S1 Fig).

The AMOVA revealed much greater variation within populations (66%) than among the populations (34%), confirming the low genetic differentiation among the subpopulations, but high genetic differentiation within subpopulations (Table 2).

**Table 2:** Summary of the analysis of molecular variance (AMOVA) within and among *S*. *marianum* populations.

Source	Degree of Freedom	Sum of Square	Mean Sum of Square	Estimated Variance	Percentage Variation
**Among Pop.**	2	76385,5	38192,8	1312,0	34%
**Within Pop.**	91	235348,9	2586,3	2586,3	66%
**Total**	93	311734,4	-	3898,3	100%

### Phenotypic differentiation based on genetic classification

**Fig 4:**
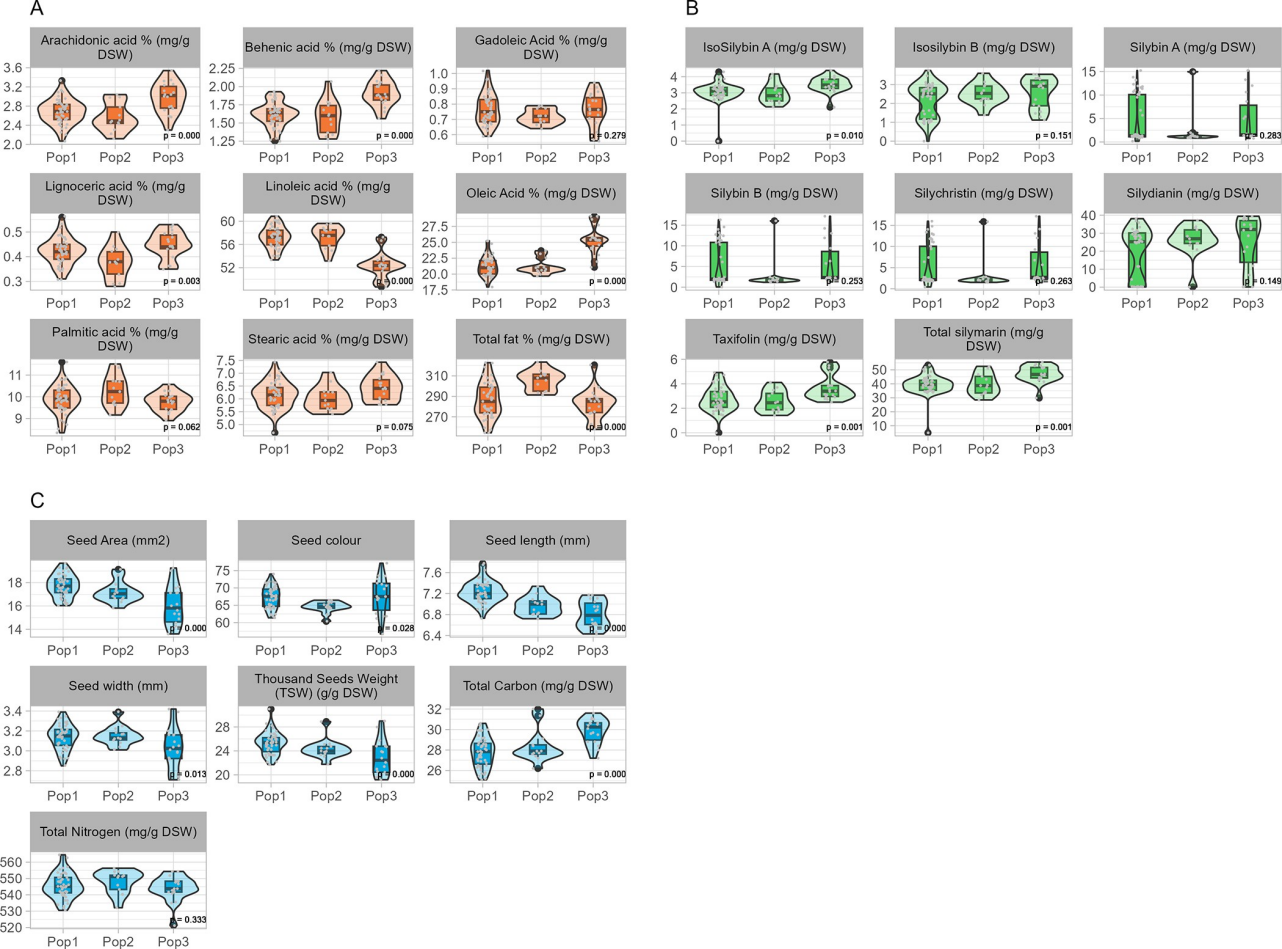
Phenotypic distribution of oil constituents (A), silymarin components (B) and fruit morphological parameters (C), among the three identified groups (Pop1, Pop2, and Pop3) of *S*. *marianum* collection. Boxplots represent the distribution of each trait, with the central line indicating the median, the box edges representing the first and third quartiles, and the whiskers extending to 1.5 times the interquartile range. Outliers are represented by individual black points. p-values are displayed below each boxplot.

Phenotypic diversity for quality traits such as silymarin and oil constituents, seed morphological parameters and other agronomic-relevant traits was previously investigated [5] as detailed in S1 Table. Here, we assessed the relatedness of the identified population structure with the measured traits (Fig 4).

Pop3, constituted by accessions from Southern Europe and mainly from Italy, was characterized by a higher percentage content of oleic acid (p-value < 0.05), arachidonic acid (p-value < 0.05), behenic acid (p-value < 0.05), stearic acid (p-value = 0.068) and lignoceric acid (p-value < 0.05); and by lower levels of linoleic acid (p-value < 0.05) than the other two populations (Fig 3A). Interestingly, Pop2, characterized by Canadian, Polish, and Belgian accessions, showed a higher total fatty acid content (p-value < 0.05) and palmitic acid (p-value < 0.05) than the other two populations. Moreover, no significant difference within the three populations was observed for the percentage content of gadoleic acid (p-value = 0.282) (Fig 4A).

In terms of flavonolignans content, a wide variability was observed among each constituent [5]. The Pop3 is characterized by higher levels of total silymarin (p-value < 0.05), taxifolin (p-value < 0.05) and Isosilybin A (p-value < 0.05), compared to the other populations (Fig 3B). No significant difference was observed within the three populations for silycristin content (p-value = 0.213), silydianin content (p-value = 0.122), silybin A (p-value = 0.227) and B (p-value = 0.202) content (Fig 4B), among these a positive correlation was previously reported [5].

The evaluation of agronomic and seed morphological traits in the frame of the population structure revealed that Pop3 showed a higher content of carbon (p-value < 0.05), compared to the other population (Fig 3C). Pop3 is characterized by a lower value of thousand seed weight (TSW) (p-value < 0.05), seed area (p-value < 0.05), seed width (p-value < 0.05), and seed length (p-value < 0.05). Interestingly, Pop2 is significantly different from Pop1 and Pop3 for the seed color (p-value < 0.05) (Fig 3C).

Overall, the 3 populations classified with DArTseq markers showed different phenotypic means for many of the traits previously measured. Interestingly, the highest phenotypic variability in terms of both oil and silymarin content was found in Pop3 compared to Pop1 and Pop2, suggesting that an environmental selective pressure may have caused these phenotypes to be more favorable in Italy.

### Identification and annotation of outlier DArTs

**Fig 5:**
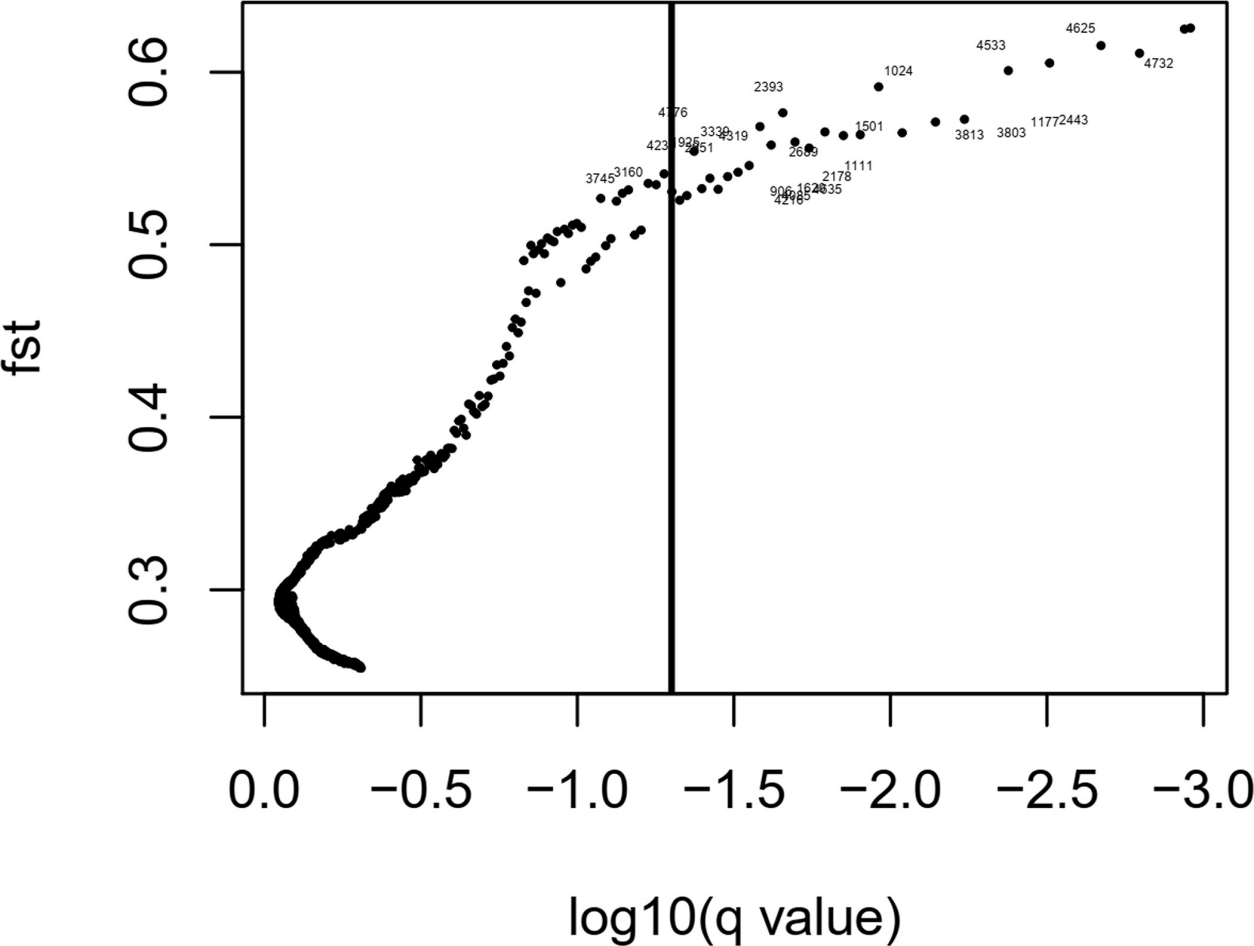
Results of the Bayescan 1.2 outlier test. Posterior probability significance threshold (vertical bar) of 0.90 after Bonferroni correction (a = 0.05). The locus number ID assigned by Bayescan 1.2 to each marker is reported only for putative outliers SNP.

Bayescan analysis detected 22 outlier loci when the three *a priori*-defined populations were compared (Fig 5). The outlier SNPs showed a F_ST_ threshold of 0.18 (FDR q-value < 0.05) and spanned chromosomes 1B, 2A, 2B, 3A, 3B, 5A, 5B, 6B, 7A, and 7B (Table 3 and S3 Table). Among them, 17 were located within annotated genes (Table 3). The highest number of annotated genes were found on chromosomes 5 and 10, followed by chromosome 1, whereas the lowest were found on chromosomes 8, 13, 14 and 17 (Table 3). Eight outlier SNPs showed higher frequency (Allele frequency > 0.9) in Pop1 and Pop2 compared to Pop3 (Allele frequency < 0.2), whereas fourteen different markers were almost fixed in Pop3 (Allele frequency > 0.9) but not in Pop1 and Pop2 (Allele frequency < 0.2) (Table 3), suggesting that a certain selective pressure might exist within *S*. *marianum* populations.

**Table 3:** Frequency alleles of outlier SNPs detected by Bayescan in the three *S*. *marianum* groups. The DArT ID, chromosome, position and candidate genes were also provided. DArTs with allele frequencies (AF) ≤ 0.5 were scored in orange, whereas those with AF ≥ 0.5 were plotted in green.

DArT locus ID	Chr	pos	Pop1	Pop2	Pop3	*S*. *marianum* gene ID	Best hit (Blast tool in NCBI)
**5874342**	chr01	5660432	0.040	0.011	0.923	Smar01g005980	*C*. *cardunculus* var. scolymus S-adenosylmethionine uptake transporter-like (LOC112507931)
**5871899**	chr01	5694193	0.961	0.991	0.013	-	-
**5870893**	chr01	59370616	0.039	0.009	0.987	Smar01g045370	*C*. *cardunculus* var. scolymus serine/threonine-protein kinase Nek1-like.
**21306630**	chr01	54632916	0.040	0.011	0.923	-	-
**5871961**	chr03	44458124	0.905	0.988	0.046	Smar03g031300	*C*. *cardunculus* var. scolymus AT-hook motif nuclear-localized protein 10-like (AHL gene family) [56]
**5871447**	chr03	51265377	0.104	0.011	0.956	-	
**5869938**	chr03	6730720	0.055	0.010	0.927	Smar03g006250	*C*. *cardunculus* var. scolymus TBCC domain-containing protein 1 (LOC112512596) [53]
**5872040**	chr03	39722574	0.906	0.991	0.013	-	-
**5871235**	chr05	36436699	0.922	0.989	0.046	-	-
**5870307**	chr05	11568441	0.063	0.008	0.986	Smar05g013800	*C*. *cardunculus* var. scolymus protein LYK2 (LOC112510657) [54]
**5871854**	chr05	11837972	0.051	0.011	0.958	Smar05g014220	*C*. *cardunculus* var. scolymus mitogen-activated protein kinase kinase 3 (LOC112518132)
**5871636**	chr05	45336151	0.053	0.010	0.957	Smar05g040540	*C*. *cardunculus* var. scolymus protein Chromatin Remodeling 20 (LOC112523559) [52]
**5873463**	chr08	33292851	0.950	0.990	0.014	Smar08g023500	*C*. *cardunculus* ATP-dependent Clp protease ATP-binding subunit ClpC (clpC)
**5871896**	chr10	14109277	0.016	0.007	0.933	Smar10g015040	*C*. *cardunculus* var. scolymus protein WVD2-like 7
**5874265**	chr10	148361	0.925	0.950	0.015	Smar10g000170	*C*. *cardunculus* var. scolymus eukaryotic translation initiation factor 2 subunit alpha homolog (LOC112501554)
**5871972**	chr10	150074	0.089	0.049	0.985		
**5870087**	chr10	13621612	0.028	0.010	0.901	Smar10g014580	*C*. *cardunculus* var. scolymus fructokinase-like 2
**5869787**	chr11	21957511	0.040	0.009	0.958	Smar11g015190	*H*. *annuus* exopolyphosphatase PRUNE1-like
**5873408**	chr11	28681801	0.056	0.010	0.958	Smar11g021760	*C*. *cardunculus* var. scolymus putative vesicle-associated membrane protein 726
**5869914**	chr13	32006287	0.066	0.165	0.983	-	-
**5874164**	chr14	4509403	0.755	0.988	0.015	Smar14g003320	*C*. *cardunculus* var. scolymus Protein transport SEC20 (uncharacterized LOC112518201)
**5874584**	chr17	21403322	0.667	0.986	0.015	Smar17g015100	*C*. *cardunculus* var. scolymus LRR receptor-like serine/threonine-protein kinase FEI 1

The gene function of the identified outliers located within annotated genes was inferred using the best-hit approach through the Blast. Several outliers were found in *S*. *marianum* genes mainly encoding for signaling proteins and transporters involved in biological functions or primary metabolism-related functions [52–56]. Interestingly, the DArT “5872821” was found within the gene *Smar02g039390*, the putative ortholog of *CcPHR1-like* which encodes for a phosphate starvation response regulator in conditions of limited phosphorus availability [55], a gene family characterized in diverse genera of the family Gramineae that can be linked to selection under diverse environmental conditions.

The DArT “5874342” matched with *Smar01g005980*, orthologous to *S-adenosylmethionine uptake transporter*, whereas the DArT “5870307” was found spanning the gene *Smar05g013800*, orthologs of *Receptor-Like Kinase 2* (*LKY2*) encoding gene which is involved in elicitor—mediated biotic responses [54]. Furthermore, the DArT “5869938” falls into *Smar03g006250*, a gene locus encoding for TBCC domain-containing proteins, known to be involved in organ development and vascularization in diverse plant species [53]. The gene locus Smar05g040540 associated with the DArT marker “5871636”, according to the function of its putative ortholog in *Drosophila melanogaster* [52], could be also involved in plant environmental adaptation.

### Allelic effect of DArT under natural selection on different phenotypic traits

**Fig 6:**
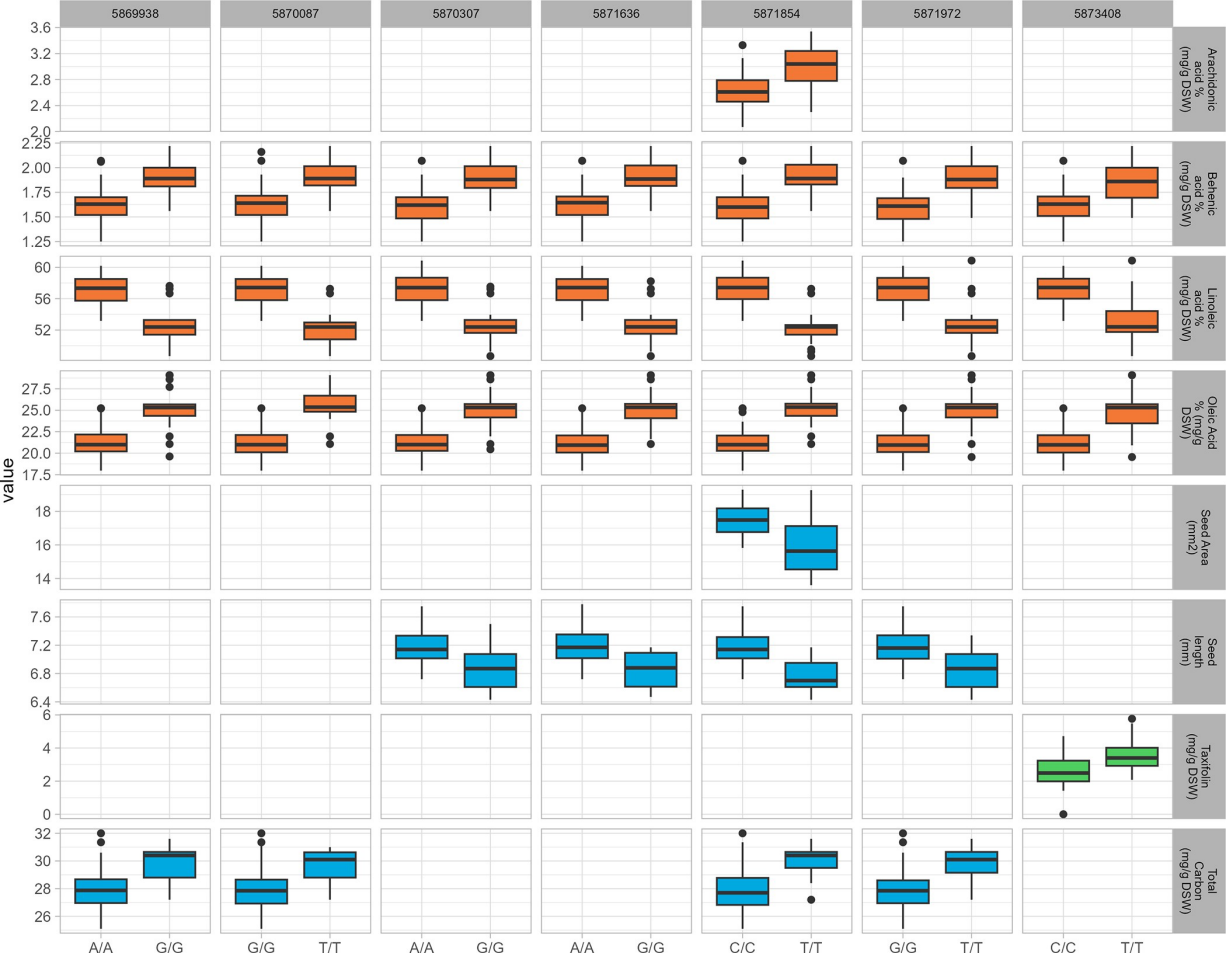
Boxplot of DArTs putatively under natural selection with significant effects (p-value < 0.0001) on phenotypic traits of oil constituents in orange, fruit morphological parameters in blue, and silymarin components in green. For each selected DArT, the germplasm lines were divided into two groups according to their genotypic state (homozygous for reference or alternate allele). The X-axis represents the two alleles for each DArT, while the Y-axis corresponds to the mean of the selected phenotypic trait. Boxplots represent the distribution of each trait, with the central line indicating the median, the box edges representing the first and third quartiles, and the whiskers extending to 1.5 times the interquartile range. Outliers are represented by individual points.

Among the DArT identified by Bayescan (Table 3), seven loci with high allele frequency (>0.9) in Pop3 showed a significant effect (p-value < 0.0001) on linoleic and oleic acid contents (Fig 6). Interestingly, the favourable haplotype for higher linoleic acid content (AGAACGC) was almost fixed in Pop3 (81.48%), whereas the unfavourable haplotype (GTGGTTT) abundant in both Pop1 (79.24%) and Pop2 (95.85%) (S4 Table). In addition, an opposite trend was observed for the same haplotype for oleic and behenic acid content (Fig 5), confirming the negative relationship between oleic and linoleic acid content.

Four loci (“5871854”, “5870087”, “5869938” and “5871972”), all shared with those identified for linoleic and oleic acid contents, were identified as significant also for total carbon content, with favourable haplotype (GTTT) almost fixed in Pop3. Whereas four loci (“5870307”, “5871854”, “5871636” and “5871972”) slightly impacted seed length, with the favourable haplotype (AACG) being almost fixed in Pop1 and Pop2 but not in Pop3.

Among the selected DArTs, “5871854” was the only one showing a significant low impact on seed area and arachidonic acid content, whereas the locus “5873408” slightly impacted the taxifolin content.

## Discussion

This study examined the genetic structure of an *ex-situ S*. *marianum* collection through both parametric and non-parametric approaches and indicated that individuals could be grouped into three distinct groups (Pop1-3), largely reflecting their geographical origins. Unespectively, in some instances, samples derived from different plants belonging to the same accession clustered in different groups suggesting that although being kept and reproduced in different GenBank, the accessions may still have residual heterogeneity as originally collected from the wild. Interestingly, accessions with non-variegated leaves (G5, G17, and G18) were included in Pop2 except SIL3 (*S*. *eburneum* accession) which, despite the non-variegated leaves, grouped into Pop1. This unexpected result suggested a misclassification of SIL3 as *S*. *eburneum* by the original seed collector, likely due to the absence of variegation on its leaves. Despite previously stated [4], the absence of leaf variegation is not a distinctive feature of *S*. *eburneum* [4], given that this trait is not mentioned in the botanical description of the species [1]. A more in-depth analysis, encompassing both genetic and phenotypic characterization of additional *S*. *eburneum* accessions will clarify the classification of SIL3, allowing us to better define the species classification in the *Silybum* genus, being more likely an *S*. *marianum* accession. Given the geographical clustering obtained here and considering that *S*. *marianum* is only naturalized in North and South America, New Zealand, and Australia [57], we may hypothesise that the species has been introduced to Canada from Poland. A suggestive hypothesis that should be validated with larger datasets.

Comparing these results with the phenotypic ones reported by Martinelli et al. [5] on the same genetic materials, the discriminating power of the SNP markers was highlighted. Effectively, Martinelli et al. [5] did not identify distinctive groups in the same *S*. *marianum* collection by using morphological and biochemical data only (e.g. fruit morphology, total oil content, oil fatty acid profile, taxifolin, flavonolignans content), with Italian accessions (Pop3) being distributed across three out of nine distinct clusters. This clustering analysis effectively distinguished between accessions with silymarin chemotypes A and B. Specifically, five clusters grouped genotypes with chemotype A, while another three grouped those with chemotype B. On the contrary, the clustering based on genomic markers is not able to separate the different silymarin chemotypes (S5 Table). This could suggest that this important phenotypic trait, known to be genetically inherited [58], is probably not associated to the phyletic origin of the accessions, but unevenly spread in world germplasm. These findings contrasted with Shokrpour et al. [59], who clustered the accessions based on their origin, by analyzing the morphological characteristics and flavonolignans properties of 32 milk thistle ecotypes, collected from northern and southern regions of Iran; suggesting in this case a differentiation probably associated to the different geomorphology of the Iranian regions.

Grouping milk thistle accessions based on their geographic origins by using molecular markers was also confirmed by Mohammadi et al. [23]. The authors used AFLP markers to assess the molecular diversity in 32 populations of *S*. *marianum* collected from seven provinces of Iran and identified three major groups consistent with their geographical grouping, with only a few exceptions. Correspondence between genetic and geographical distance was also reported for other plants such as *C*. *odorata* specimens [60] using DArT SNP markers. The authors adopted a target capture method coupled with short-read sequencing to identify spatially informative SNPs that differentiate species based on latitude, temperature, and precipitation.

Regarding the diversity indices considered in this study, Nei’s genetic diversity (H, 0.17) and Shannon diversity Index (I, 0.27), our results are consistent with those obtained by Mohammadi et al. [23], where H and I were 0.20 and 0.29, respectively, and by Saghalli et al. [24], where H and I were 0.33 and 0.49, respectively. In contrast, these results differ from those of Rafizadeh et al. [22], where the average H was 0.72 and the average I was 0.83. Specifically, Rafizadeh et al. [22] investigated 80 *S*. *marianum* genotypes from 8 populations in Iran. Although their H and I values are higher, they also found greater genetic diversity than in our study, which seems to be attributable to within-group (58%) rather than between-group variation (42%). The same authors highlighted that various factors, including genetic drift, mutation, and natural selection, along with genetic marker systems, could impact genetic differentiation [22].

Based on pairwise fixation index (F_ST_), Pop3 showed a greater differentiation compared to the other subpopulations, consistent with other diversity indices such as private alleles and heterozygosity. The higher differentiation of Pop3 is notably evident when observing the phenotypic distribution based on genetic clustering, since a divergent pattern for oleic, arachidonic, behenic, and linoleic acid content, was observed compared to the other two groups. The oil content found in Pop3 is comparable with that identified in the five species most used for oil production (e.g., sunflower, peanut, rapeseed, mustard, and olive oil) and cultivated in Eastern Europe suggesting that milk thistle, could also be a viable vegetable oil source [61]. Pop3 also exhibited a higher total carbon content and smaller seed area, width, and length, all important phenotypic traits important for future breeding programs. However, it is important to note that Italian accessions abounded in our collection, thus probably this factor might have an impact.

Therefore, although further studies are needed, Bayescan analysis, a widely used method for detecting loci under selection [49], allowed us to identify seven loci fixed within Pop3 and probably influencing the phenotypic traits described above. This opens an interesting scenario where beneficial identified haplotypes might be the basis for developing milk thistle lines with higher levels of oleic, arachidonic, and behenic acids, and lower levels of linoleic acid, paving new avenues for enhancing the nutritional and agronomic characteristics of milk thistle. For instance, Pearson et al. [62] used the Bayescan method to identify SNPs associated with changes in foliar water-soluble carbohydrate levels in 935 *Trifolium repens* L. individuals. Among the 33 SNPs detected, one was found within the intron of *ERD6-like 4*, a gene encoding a sugar transporter on the vacuole membrane, prompting further investigation into these genomic regions. Additionally, a recent study on *Helichrysum italicum* (Roth) G. Don led to the identification of four AFLPs strongly associated with the bioclimatic variables [63], offering Asteraceae breeders an opportunity to enhance various traits through marker-assisted selection. Indeed, incorporating genetic material from individuals carrying the selected loci into milk thistle breeding populations can potentially enhance desired traits, especially using Italian accessions (Pop3) as donors. However, given the population size, it will be important to strengthen our results with molecular validations and with the *de novo* sequencing of a higher number of accessions, thus providing a deeper understanding of the genetic basis of important traits, and enhancing the success of breeding programs for milk thistle.

## Conclusions

Understanding the genetic diversity of minor species such as *S*. *marianum*, still partially domesticated and little studied, is a fundamental step in exploiting their genetic resources. It also plays a significant role in designing efficient plant breeding programs and determining which genotypes to cross for developing new populations. The present study indicates that there is potential to enhance milk thistle for desirable traits through genetic variation. DArTseq has proven to be a robust and proficient tool to produce large numbers of informative markers that reveal a population structure and genetic differentiation in our germplasm collection. A total of twenty-two markers were identified as putatively under natural selection. Among these, seven SNP markers probably exerted significant effects on various phenotypic traits. These marker SNPs, if appropriately validated, represent a good tool for starting a milk thistle breeding aimed at expanding the use of the plant for food and non-food uses.

## Supporting information

S1 FigBarplot showing the mean and standard deviation as error bars of allelic patterns for codominant data.The figure displays the following parameters: unbiased expected heterozygosity $uHe=(\frac{2N}{\left(2N-1\right)})\cdot He$; the number of private alleles unique to a single population; the number of locally common alleles found in 50% and 25% or fewer populations; number of effective alleles $Ne=\frac{1}{\left(\sum \cdot {pi}^{2}\right)}$; number of different alleles with a frequency > = 5%; number of different alleles (Na); Shannon’s information index *I* = −1⋅∑(*pi*⋅*Ln*(*pi*)); and the expected heterozygosity *He* = 1−∑⋅*pi*^2^.(TIF)

S1 TablePhenotypic data.List of parameters/features displayed: AWclust_K3, PCA_K3, Admx_K3, Taxifolin content (mg/g DSW), Total silymarin content ($\frac{mg}{g}DSW$), Silychristin content ($\frac{mg}{g}DSW$), Silydianin content ($\frac{mg}{g}DSW$), Silybin A content ($\frac{mg}{g}DSW$), Silybin B content ($\frac{mg}{g}DSW$), IsoSilybin A ($\frac{mg}{g}DSW$), Isosilybin B ($\frac{mg}{g}DSW$), Total Carbon content ($\frac{mg}{g}DSW$), Total Nitrogen content ($\frac{mg}{g}DSW$), Thousand Seeds Weight (TSW) ($\frac{g}{g}DSW$), Seed Area (*mm*^2^), Seed length (*mm*), Seed width (*mm*), Seed colour measured as the mean of gray tones, Total fat content % ($\frac{mg}{g}DSW$), Palmitic acid % ($\frac{mg}{g}DSW$), Stearic acid % ($\frac{mg}{g}DSW$), Oleic Acid content % ($\frac{mg}{g}DSW$), Linoleic acid % ($\frac{mg}{g}DSW$), Arachidonic acid % (mg/g DSW), Gadoleic Acid % ($\frac{mg}{g}DSW$), Behenic acid % ($\frac{mg}{g}DSW$), Lignoceric acid % ($\frac{mg}{g}DSW$).(CSV)

S2 Table Number of DArT markers for each scaffold. (CSV)

S3 TableSummary of heterozygosity, f-statistics, and polymorphism by population for codominant data.The table displays the following parameters: sample size (N); number of different alleles (Na); number of effective alleles $Ne=\frac{1}{\left(\sum \cdot {pi}^{2}\right)}$, Shannon’s information index *I* = −1⋅∑(*pi*⋅*Ln*(*pi*)); observed $Ho=\frac{N_{Hets}}{N}$, expected *He* = 1−∑⋅*pi*^2^, and unbiased expected heterozygosity $uHe=(\frac{2N}{\left(2N-1\right)})\cdot He$; fixation index $F=\frac{\left(He-Ho\right)}{He}=1-\frac{Ho}{He}$.(CSV)

S4 Table Genetic profile of seven DArT markers potentially under selection was shown for each population and DArT sample code. (CSV)

S5 Table Summary Table of individual plant chemotype among the three identified groups (Pop1, Pop2, and Pop3) of the *S*. *marianum* collection. (CSV)
