# Supplementary figures and images for: Genetic diversity of a Silybum marianum (L.) Gaertn. germplasm collection revealed by DNA Diversity Array Technology (DArTseq)
Source: PLoS One. 2024 Aug 7;19(8):e0308368. doi: 10.1371/journal.pone.0308368 (PMC11305583; doi:10.1371/journal.pone.0308368)

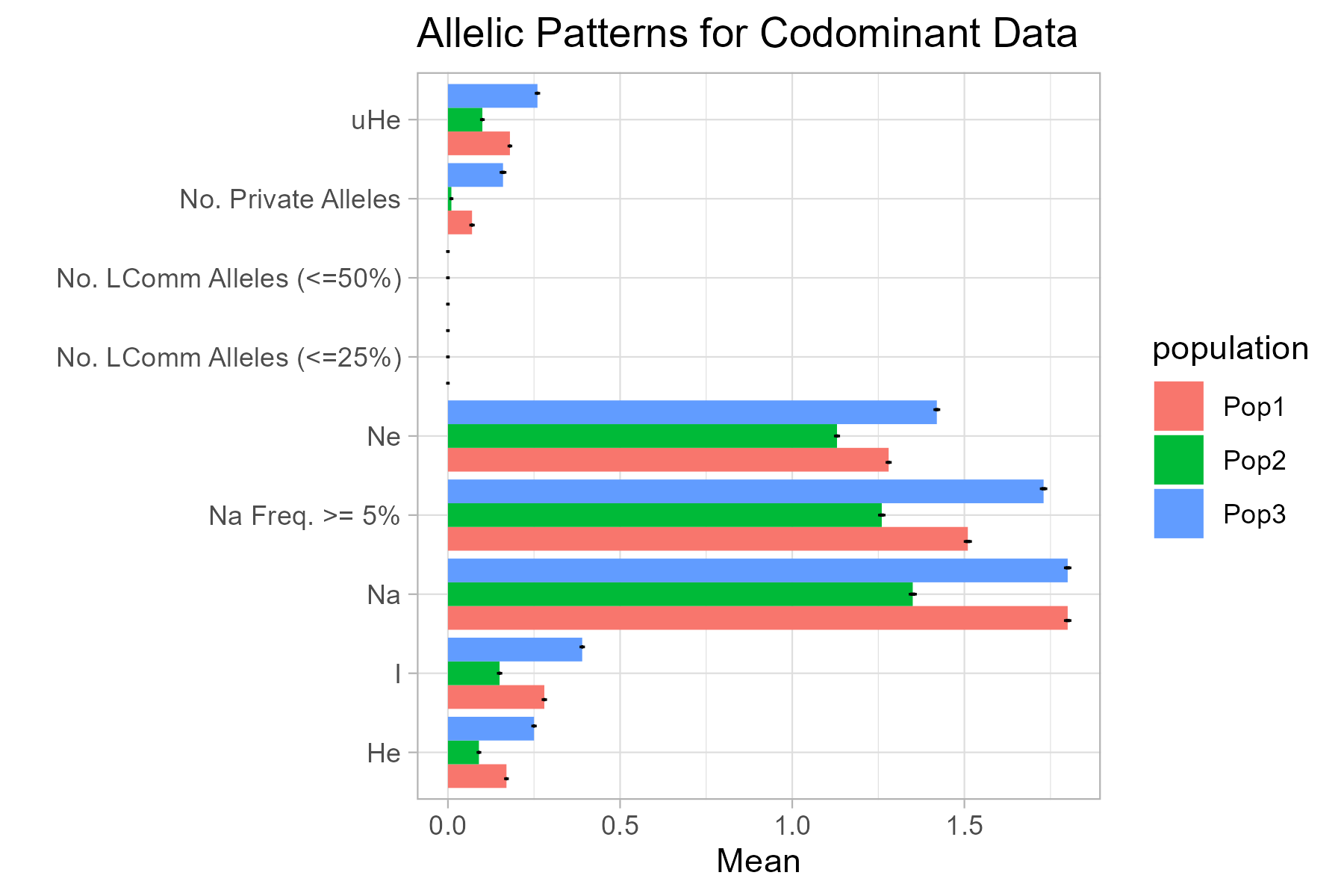

Supplement: S1 Fig — The figure displays the following parameters: unbiased expected heterozygosity uHe=(2N(2N−1))⋅He​; the number of private alleles unique to a single population; the number of locally common alleles found in 50% and 25% or fewer populations; number of effective alleles Ne=1(∑⋅pi2)​; number of different alleles with a frequency > = 5%; number of different alleles (Na); Shannon’s information index I = −1⋅∑(pi⋅Ln(pi)); and the expected heterozygosity He = 1−∑⋅pi2. (TIF) [file pone.0308368.s001.tif]
